# Deep learning-based overall survival prediction model in patients with rare cancer: a case study for primary central nervous system lymphoma

**DOI:** 10.1007/s11548-023-02886-2

**Published:** 2023-04-21

**Authors:** Ziyu She, Aldo Marzullo, Michela Destito, Maria Francesca Spadea, Riccardo Leone, Nicoletta Anzalone, Sara Steffanoni, Federico Erbella, Andrés J. M. Ferreri, Giancarlo Ferrigno, Teresa Calimeri, Elena De Momi

**Affiliations:** 1 Department of Electronics, Information and Bioengineering, Politecnico di Milano, Milan, Italy; 2 Department of Mathematics and Computer Science, University of Calabria, Rende, Italy; 3 Department of Experimental and Clinical Medicine, University of Catanzaro, Catanzaro, Italy; 4 Neuroradiology Unit, IRCCS San Raffaele Scientific Institute, Milan, Italy; 5 Lymphoma Unit, IRCCS San Raffaele Scientific Institute, Milan, Italy

**Keywords:** PCNSL, OS prediction, Deep learning, Model explainability, Grad-CAM

## Abstract

**Purpose:**

Primary central nervous system lymphoma (PCNSL) is a rare, aggressive form of extranodal non-Hodgkin lymphoma. To predict the overall survival (OS) in advance is of utmost importance as it has the potential to aid clinical decision-making. Though radiomics-based machine learning (ML) has demonstrated the promising performance in PCNSL, it demands large amounts of manual feature extraction efforts from magnetic resonance images beforehand. deep learning (DL) overcomes this limitation.

**Methods:**

In this paper, we tailored the 3D ResNet to predict the OS of patients with PCNSL. To overcome the limitation of data sparsity, we introduced data augmentation and transfer learning, and we evaluated the results using *r* stratified *k*-fold cross-validation. To explain the results of our model, gradient-weighted class activation mapping was applied.

**Results:**

We obtained the best performance (the standard error) on post-contrast T1-weighted (T1Gd)—area under curve $=0.81(0.03)$, accuracy $=0.87(0.07)$, precision $=0.88(0.07)$, recall $=0.88(0.07)$ and *F*1-score $=0.87(0.07)$, while compared with ML-based models on clinical data and radiomics data, respectively, further confirming the stability of our model. Also, we observed that PCNSL is a whole-brain disease and in the cases where the OS is less than 1 year, it is more difficult to distinguish the tumor boundary from the normal part of the brain, which is consistent with the clinical outcome.

**Conclusions:**

All these findings indicate that T1Gd can improve prognosis predictions of patients with PCNSL. To the best of our knowledge, this is the first time to use DL to explain model patterns in OS classification of patients with PCNSL. Future work would involve collecting more data of patients with PCNSL, or additional retrospective studies on different patient populations with rare diseases, to further promote the clinical role of our model.

## Introduction

Primary central nervous system lymphoma (PCNSL) is a rare, aggressive form of extranodal non-Hodgkin lymphoma confined to the central nervous system, including the brain, cerebrospinal fluid and eyes without evidence of systemic spread [1]. The treatment of patients with PCNSL includes two phases, induction and consolidation. Induction consists of high-dose methotrexate-based chemotherapy, while whole-brain radiotherapy (WBRT) is a consolidation option in patients with a disease response to induction [2]. However, only about $56\%$ of patients are eligible for the consolidation and late neurotoxicity is an important issue especially when WBRT has been used [3]. The reason of this poor response to treatment remains unknown. Nevertheless, to predict the treatment response in advance is still of paramount importance as to better understand tumor behavior and aid clinical decisions. The overall survival (OS), the length of time from either the date of diagnosis or the start of treatment for a disease to the last follow-up or eventually death, is the gold standard primary endpoint to evaluate prognosis [4]. Nowadays, radiomics, a computational tool to extract high-dimensional features from medical images, has been popular in customizing treatment of oncology, powered by the development machine learning (ML) [5, 6]. However, ML requires suitable parameters, while a large number of radiomic features is extracted from images; thus, an adequate feature selection must be performed.

Deep learning (DL), which directly extracts features from the images, has emerged as one of the most powerful technologies in the past decade. It has boosted the progress in the field of computer vision [7]. In fact, DL models are data hungry due to the stack of hidden layers. In the case of small dataset, training a very deep network would lead to overfitting. Data augmentation, transfer learning and cross-validation are common methods to alleviate the problem of small dataset and overfitting. Furthermore, most existing work uses cross-validation to predict model uncertainty, which can be referred to artificial intelligence (AI) safety [8, 9]. In addition, it is important to interpret DL-based results to provide intuitive outputs for human review, thus, assess AI safety and accelerate the adoption of DL into the real-world medical application [10–12]. However, there are few studies to implement DL in the analysis of PCNSL while analyzing model uncertainty and explainability.

In this paper, a DL model 3D ResNet was used to perform binary OS classification of patients with PCNSL. Then, Gradient-weighted Class Activation Mapping (Grad-CAM) was tailored to visualize and explain the patterns of our model. Specifically, our main contributions were the following:

1. Training from scratch vs transfer learning: We evaluated the 3D ResNet on different MRI modalities under training from scratch and transfer learning, respectively, and obtained one specific MRI modality with the best performance, which is consistent with the clinical outcome.
2. Uncertainty prediction and pattern explainability: To the best of our knowledge, this is the first trial to output the uncertainty and success and failure patterns of the 3D DL model in OS classification of patients with PCNSL, which can be referred to AI safety in the medical field.

## Related work

Currently, radiomics-based ML has taken advantage of magnetic resonance imaging (MRI). ML in training classifiers to distinguish between glioblastoma and PCNSL performed well with respect to area under curve (AUC) [13, 14]. However, this type of studies is limited to the differentiation of PCNSL from glioblastoma. Besides, ML requires manual feature extraction from images, which is time- and resource-consuming. It is also found that some feature extraction methods would introduce too much noise and lead to ML model unstability [15]. A famous example is that none of radiomics-based ML approaches is able to outperform the age-only baseline with cross-validation in the BraTS survival prediction challenge 2018 [16].

On the contrary, DL has been applied in medical images of brain diseases and obtained state-of-the-art results, with minimal prior medical knowledge and feature selection. A DL model containing stacked auto-encoders and a softmax output layer was designed to perform diagnosis of Alzheimer’s disease, and a significantly better performance (accuracy is 0.88) was achieved compared with support vector machine (accuracy is 0.84) [17]. A 3D U-Net for brain tumor segmentation using separable 3D convolutions achieved competitive results with Dice scores of 0.69, 0.84 and 0.78 for the enhancing tumor, the whole tumor and the tumor core, respectively [18]. These studies may have great potential to lead to a new perspective for computational medical research. However, to achieve state-of-the-art accuracy, DL models require large labeled dataset for training, which it is usually prohibitive to be acquired due to the extensive annotation efforts and the demand of expertise in the medical domain.

In the case rare tumors, there have been successful examples tackling the problem of small dataset. An EfficientNet-based convolution neural network (CNN) was used to classify primary bone tumors from preoperative radiographs with better accuracy than junior radiologists; data augmentation and cross-validation were introduced to mitigate the issue of small dataset and predict model uncertainty [19]. Low-shot DL was adopted for the detection of conjunctival melanoma on ocular surface images, and the MobileNetV2 trained under transfer learning and with generative adversarial network (GAN)-based augmentation displayed the highest accuracy of 0.97 [20]. However, there are seldom attempts to explain the patterns of the DL models in rare tumors.

## Method

### Dataset and image preprocessing

**Fig. 1:**
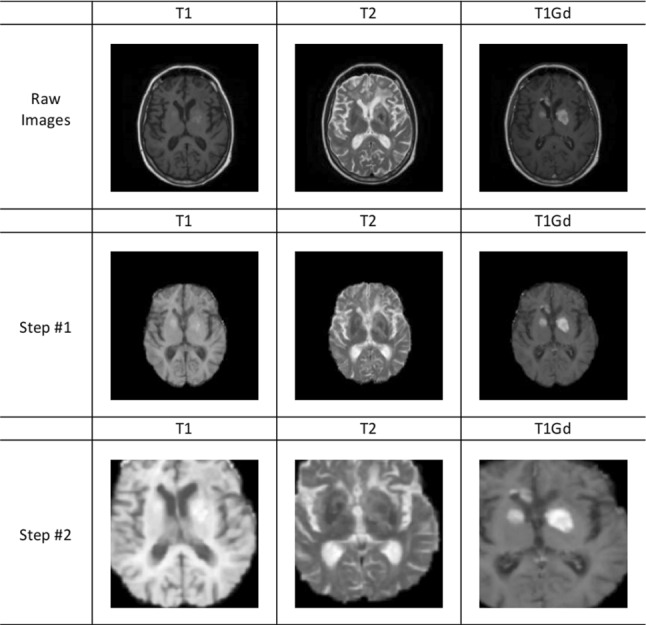
Preprocessing steps of MRI of patients with PCNSL

Multi-center brain MRI modalities with different scanners (Philips Medical System Achieva and Whole, GE Medical System/Optima MR450 and SIEMENS AERA) of 56 patients with PCNSL between January 2010 and November 2019 were retrospectively collected at San Raffaele Hospital in Milan (Italy). Different MRI modalities, namely T1-weighted (T1), T2-weighted (T2) and post-contrast T1-weighted (T1Gd), which are helpful for diagnosing different neurological pathology, were collected. Also, clinical data (the age, the level of lactate dehydrogenase, the cerebrospinal fluid total protein, the performance status, the IELSG score and the OS) of these patients was collected. In particular, the OS is positive means the OS is more than 1 year and the OS is negative means the OS is less than 1 year; in our dataset, there are 30 positive cases and 26 negative cases, respectively. Differences among these three MRI modalities are depicted in Fig. 1.

We followed standard image preprocessing techniques—bias field correction, registration, skull stripping, intensity normalization, and voxel resampling using 3D Slicer [21, 22]. Furthermore, we performed additional preprocessing, including background removing and data augmentation, to enhance the stability of the model. In fact, these images were collected from different hospitals and with different scanners, which would induce unavoidable systemic noise. In particular, the following steps were performed (Fig. 1):

1. Step #1: The N4ITK MRI bias correction module was used for bias field correction. All MRI acquisitions for each patient were registered on T1Gd. The Z-score method was used to normalize image intensity by subtracting the mean of the image and then dividing by the standard deviation of all the voxels in the image. The size of each voxel after resampling was $1\, \hbox {mm} \times 1\ \hbox {mm} \times 1\ \hbox {mm}$.
2. Step #2: Regions around the brain were removed first to help the model focus on the foreground (brain and tumor only). For data augmentation, affine transformation, elastic deformation, random spatial cropping and random rotation were performed for each MRI modality. The size of random spatial cropping was $96 \times 96 \times 96$. The degree, the axis and the probability of random rotation were $15^{\circ }$, *x*-axis and 0.5, respectively. The image size was resized to $128 \times 128 \times 128$ for each MRI modality.

### Model architecture and transfer learning

**Fig. 2:**
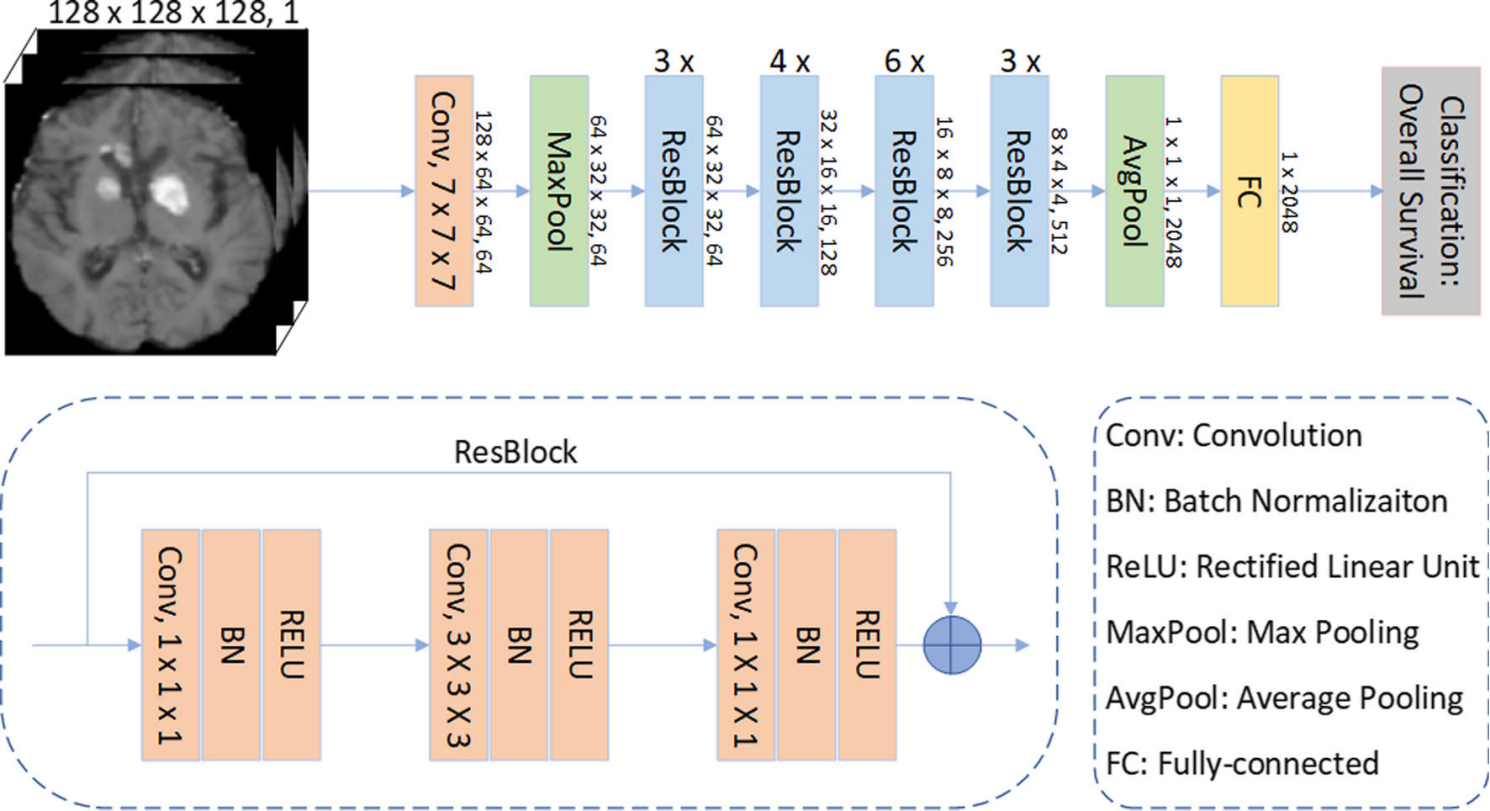
Architecture of the 3D ResNet: Input a 3D image, use the convolution layers and pooling layers as the encoder to extract features, and finally, a linear FC layer was used to output the OS classification result

The model architecture was based on the 3D voxel ResNet since our inputs are three-dimensional images (Fig. 2). Moreover, the 3D voxel network is more powerful at capturing the spatial information in natural or organic formations than its 2D version [7, 23]. Furthermore, to improve the performance of the model, transfer learning was used. First, we pre-trained the 3D ResNet on the BraTS2020 dataset for the task OS classification of patients with glioblastoma. In particular, BraTS2020 consists of multi-modal preoperative images of 235 glioblastoma patients from 19 institutions with the reported resection status and known OS [24]. Then, we fine-tuned the 3D ResNet on our PCNSL dataset for the target task OS classification of patients with PCNSL.

### Cross-validation and pattern visualization

To perform convincible classification on the small dataset and avoid overfitting, we also used *r* stratified *k*-fold cross-validation. The *r* stratified *k*-fold cross-validation reports the mean result across all iterations, and the uncertainty between the mean performance and the unknown underlying performance can be estimated using the standard deviation (std). In this paper, we set hyperparameters $r = 2$ and $k = 5$; after choosing some specific evaluation metric, we obtained the corresponding mean and std for these 10 iterations.

To visualize the patterns of our model, we customized the original Grad-CAM to fit the 3D ResNet-based architecture and output the 3D activation map of the last convolution layer on the original input based on the prediction of the model [10]. Then, we extracted the 2D activation map of the slice near the tumor.

### Experiment description

Since PCNSL is a rare cancer and it is hard to reach large dataset, we split the original PCNSL dataset into training set (45 patients) and validation set (11 patients) for each iteration of *r* stratified *k*-fold cross-validation.

To compare clinical practice with our model, we used ML models—logistic regression (LR) and support vector machine (SVM) on clinical data and radiomics data, respectively, to predict the OS of patients with PCNSL. For training from scratch (T), we trained the 3D ResNet on T1, T2 and T1Gd, respectively, to predict the OS of patients with PCNSL. The weights of the 3D ResNet were initialized using a uniform distribution [7]. For transfer learning (TL), we concatenated all MRI modalities from BraTS2020 along the channel dimension and pre-trained the 3D ResNet on these inputs and, then, fine-tuned the model on T1, T2 and T1Gd from the PCNSL dataset, respectively, to predict the OS of patients with PCNSL. In addition, for T and TL, we set a batch size of 8 and used the cross entropy as loss function. The learning rate was set to be 0.001 and was multiplied by 0.1 every 30 epochs. The Adam algorithm was used for optimization, the maximum training epoch was set to be 250, and an early stopping policy was used. The whole training process was carried out using a single NVIDIA A100 GPU (20GB). Then, we evaluated the performance of ML, T and TL: For each metric—AUC, accuracy, precision, recall and *F*1-score, we reported the corresponding mean and std. Last, we chose the model whose performance is the best evaluated in the above *r* stratified *k*-fold cross-validation process, retrained the model with the same hyperparameter setting mentioned in section “Experiment description” and output the patterns of this model.

## Results

### Quantitative analysis

In Table 1, simply using ML on clinical data, we obtained values less than 0.70 in terms of all metrics while using ML on radiomics data from T1 and T2, respectively, and we obtained larger values in terms of all metrics. For T and TL, we obtained values more than 0.70 in terms of all inputs and all metrics except for AUC. In particular, we obtained largest mean values from T1Gd under TL in terms of all metrics—AUC $$ = 0.81 (0.03)$$, accuracy $$=0.87(0.07)$$, precision $$ =0.88(0.07)$$, recall $$=0.88(0.07)$$ and *F*1-score $$=0.87(0.07)$$. We also predicted the uncertainty of the model using the std. T1Gd under TL outperforms the others while maintaining the small std 0.03–0.07 in terms of all metrics. For each metric, we used the *t* test to test whether the samples of T1Gd under TL and any other model are statistically different [25]. In Table 1, for TL, the p value for each metric is less than the significant level 0.05 except for T2 evaluated with Precision. To sum up, we observed that T1Gd under TL learns better representation of PCNSL and may help OS classification while maintaining stability in terms of all metrics.

**Table 1:** Cross-validation results of ML models and the 3D ResNet

	AUC (std)	Accuracy (std)	Precision (std)	Recall (std)	*F*1-score (std)
SVM:Clinic	0.69 (0.15)*	0.61 (0.14)*	0.62 (0.15)*	0.61 (0.14)*	0.60 (0.14)*
SVM:T1r	0.79 (0.07)	0.79 (0.07)*	0.85 (0.08)*	0.73 (0.11)	0.78 (0.08)*
SVM:T2r	0.75 (0.01)	0.76 (0.09)*	0.73 (0.09)*	**0**.**88** (0.13)	0.80 (0.08)
SVM:T1Gdr	0.56 (0.12)*	0.57 (0.11)*	0.57 (0.08)*	0.76 (0.21)	0.64 (0.11)*
T:T1	0.56 (0.04)*	0.72 (0.03)*	0.78 (0.06)*	0.73 (0.04)*	0.71 (0.03)*
T:T2	0.63 (0.18)*	0.72 (0.08)*	0.72 (0.08)*	0.71 (0.09)*	0.71 (0.09)*
T:T1Gd	0.65 (0.12)*	0.80 (0.04)*	0.81 (0.03)*	0.80 (0.03)*	0.80 (0.04)*
TL:T1	0.66 (0.12)*	0.72 (0.02)*	0.76 (0.04)*	0.72 (0.02)*	0.71 (0.03)*
TL:T2	0.69 (0.06)*	0.79 (0.04)*	0.85 (0.02)	0.79 (0.04)*	0.78 (0.05)*
TL:T1Gd	**0**.**81** (0.03)	**0**.**87** (0.07)	**0**.**88** (0.07)	**0**.**88** (0.07)	**0**.**87** (0.07)

*SVM* Support vector machine, *T* training from scratch, *TL* transfer learning. Clinic meant clinical data. T1r, T2r and T1Gdr meant radiomics data from T1, T2 and T1Gd, respectively

*Meant $p < 0.05$ in the *t* test

### Qualitative analysis

**Fig. 3:**
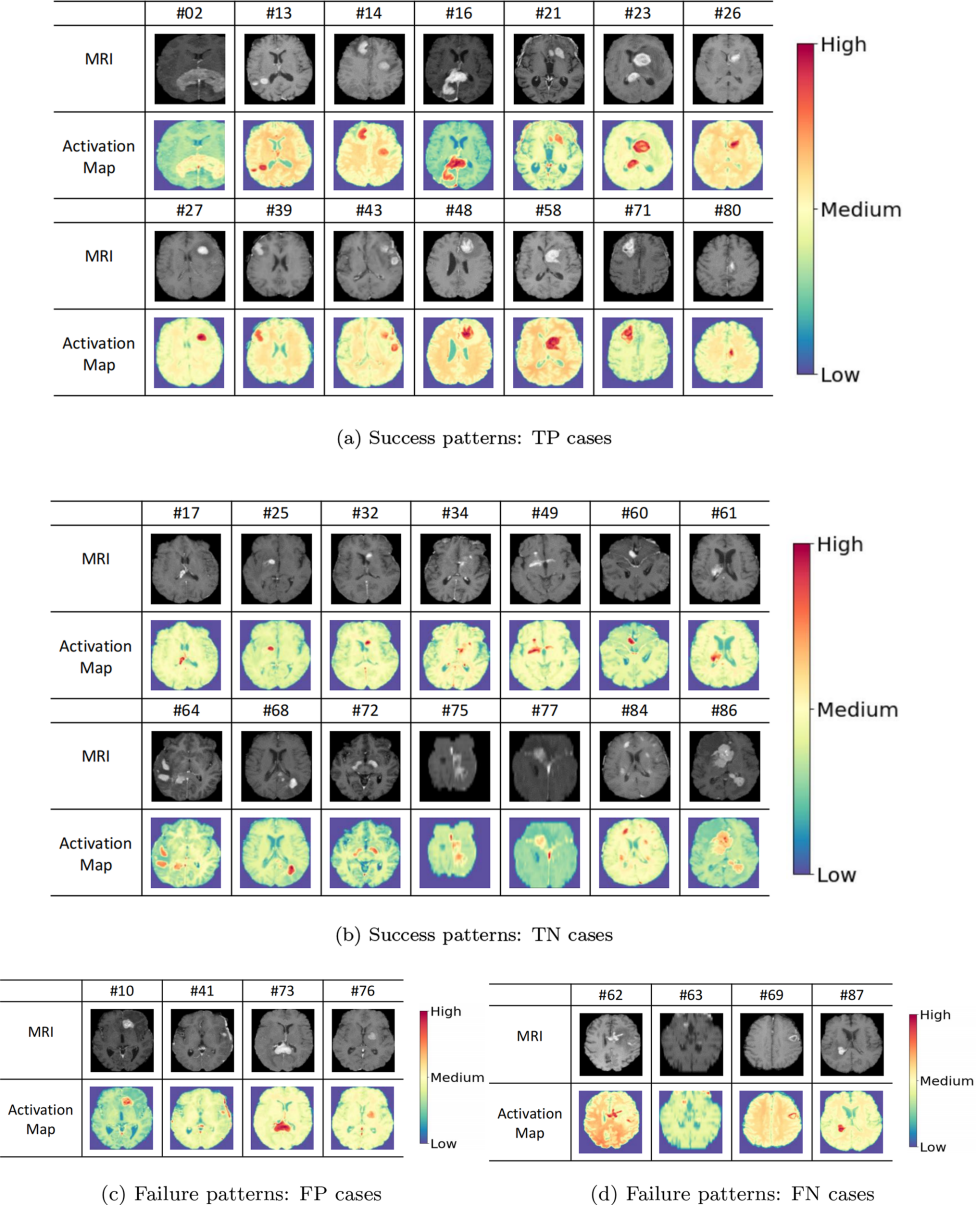
Some success and failure OS classification patterns of the 3D ResNet in PCNSL. #number was the ID of the patient. The MRI row showed the slice of TIGd near the tumor, and the activation map row showed the corresponding activation map of the slice. The colorbar on the right showed the intensity level of the activation maps

From the results in section “Quantitative analysis”, T1Gd under TL may have the best performance in OS classification of patients with PCNSL on unseen dataset. Thus, we retrained the 3D ResNet on T1Gd under TL with the same hyperparameter setting mentioned in section “Experiment description” and used Grad-CAM to analyze the corresponding success and failure patterns. The success OS classification patterns of our model are given in Fig. 3. As we can see, the model mostly focuses on the tumor region while exploring the normal part of the brain. Moreover, compared with the true-positive (TP) cases (Fig. 3a), it is more difficult to distinguish the tumor boundary from the normal part of the brain in the true-negative (TN) cases (Fig. 3b). We also gave the failure OS classification patterns: the false-positive (FP) cases (Fig. 3c) and the false-negative (FN) cases (Fig. 3d).

## Discussion

In this paper, we investigated the power of DL models in OS classification of patients with PCNSL from MRI. The OS reflects the treatment response and would help clinicians improve prognosis. Given that DL has circumvented the problem of feature selection in ML and has been widely applied in medical tasks, we further utilized its potential into the domain of PCNSL—we used the 3D ResNet to perform binary OS classification of patients with PCNSL. From the performance results, we can say that T1Gd under TL learns better representation of PCNSL, while compared with ML-based models on clinical data and radiomics data, respectively. In particular, we obtained the largest AUC 0.81(0.03) from T1Gd under TL, meaning that T1Gd under TL is stable for different threshold and the model ranks a random positive example (the OS is more than 1 year) more highly than a random negative example (the OS is less than 1 year), i.e., the model highlights the difference between the positive example and the negative example. The reason may be that T1Gd induces better contrast among brain tissues, and our model utilizes this contrast. This is consistent with previous clinical studies, where clinicians predicted the OS of patients with glioblastoma based on contrast-enhanced MRI and used AUC as a model evaluation metric [26]. In addition, the result that T1Gd under TL has the largest *F*1-score 0.87(0.07) is again consistent with previous clinical trials, where signal intensity of T1Gd has been shown to correlate with histopathology-based nuclear cell density and has been evaluated with *F*1-score [27]. Also, the value for each metric of T1Gd under TL is larger than under T, meaning that BraTS2020 does help the 3D ResNet learn subtle differences among T1Gd of patients with PCNSL. All these results confirm the stability of T1Gd under TL in the real PCNSL application. Furthermore, compared the performance between the models with *t* test, we obtained that the difference between T1Gd under TL and other models is statistically significant in most cases. In particular, for TL, the difference between T1Gd and any other model is statistically significant except for T2 evaluated with precision. This suggests that T2 would be a suboptimal choice where precision weighs more or T1Gd is missing. Also, this can be supported by the finding that precision is positively correlated with magnetic pulse duration and T2 is produced by using longer magnetic pulse duration [28]. Last, through visualizing the patterns with Grad-CAM, we observed that the model mostly focuses on the tumor region while exploring the other part of the brain, which may support the hypothesis that PCNSL is a whole-brain disease [29]. Moreover, in the cases where the OS is less than 1 year, it is more difficult to distinguish the tumor boundary from the normal part of the brain. In previous studies of glioblastoma, increasing gross total resection (GTR) rate remains a challenge to clinicians as it is difficult to distinguish the tumor boundary from normal brain parenchyma; thus, the survival rate is inferior [30, 31]. In this aspect, our model fits the regular routine of treatment of oncology. Besides, the failure patterns encourage us to include the critical clinical attributes in the future analysis of PCNSL such as the location of the tumor. This is reasonable and can be referred to AI safety in the medical field. To the best of our knowledge, this is the first time to use DL to analyze model uncertainty and explain model patterns in OS classification of patients with PCNSL. Finally, in the hope to guide future researchers, we would like to point out limitation of our study: The number of the patients is not large enough, requiring collecting a larger population to improve our model. Also, clinical data such as the location of the tumor need to be collected at the same time, to aid the decision-making procedure and enhance AI safety in PCNSL. Moreover, retrospective studies on different patient populations with rare diseases should be considered, to test the generalizability of our model and further clarify its potential clinical impact.
